# The effect of exposure to RF-EMF from the laboratory simulator of 5G NR base station on physiological parameters and cognitive abilities of male wistar rats of different ages

**DOI:** 10.1038/s41598-024-60862-5

**Published:** 2024-05-04

**Authors:** Natalia A. Krivova, Marina S. Kudabaeva, Olga B. Zaeva, Svetlana V. Borodina, Tatiana B. Lepekhina, Olga A. Pavlenko, Ramdas M. Makhmanazarov, Dmitry S. Kokin, Sergey E. Shipilov

**Affiliations:** 1 Laboratory of Experimental Physiology, Tomsk State University, Tomsk, Russia; 2 Laboratory of Neurobiology, Tomsk State University, Tomsk, Russia; 3 Department of Human and Animal Physiology, Tomsk State University, Tomsk, Russia; 4 Department of Endocrinology and Diabetology, Siberian Medical University, Tomsk, Russia; 5 Department Scientific and Educational Center “Radio Electronics Microwave”, Tomsk State University, Tomsk, Russia; 6 Department of Radiophysics, Tomsk State University, Tomsk, Russia

**Keywords:** Ecology, Ecophysiology, Risk factors

## Abstract

In this article, the impact of radiofrequency electromagnetic field (RF-EMF) exposure from a simulated base station for the 5G New Radio (5G NR) telecommunication on rats was studied. The base station affects all age groups of the population, thus, for the first time, the experiment was conducted on male Wistar rats of three different ages (juvenile, adult, and presenile). The base station exposure parameters were chosen according to ICNIRP recommendations for limiting the exposure to radiofrequency electromagnetic field: frequency 2.4 GHz with an average specific absorption rate of 0.0076 W/kg and 0.0059 W/kg over the whole body of experimental animals. Throughout the experiment, body weight was examined weekly, and the dynamics of body weight gain was monitored. Rectal and skin surface temperature on the right hind limb was monitored weekly. Testing in the Morris water maze was performed during the last, Week 5, of RF-EMF exposure. After euthanasia, organ weights were determined in experimental and control animals. None of the investigated parameters did show any statistically significant differences between exposed and control animals of the same age. The data obtained can be used to assess the possible consequences of chronic exposure to RF-EMF from 5G NR base stations.

## Introduction

5G NR (New Radio) is the next generation of wireless mobile broadband networks, a radio access technology developed by the Third Generation Partnership Project (3GPP) for the 5G (Fifth Generation) mobile network. Wireless networks are expected to support a variety of applications, such as autonomous systems and augmented/virtual reality. These diverse applications will require dense deployment of wireless networks, increasing the number of 5G base stations and 5G-enabled devices^1^. However, the widespread adoption of new technologies has led to increasing protests from the public which are caused by conflicting opinions about their safety, both for human health and for the environment as a whole. Radiofrequency electromagnetic field (RF-EMF), impacting on already existing natural or anthropogenic electromagnetic fields, is increasingly recognized as a new form of environmental pollution^2^. The International Agency for Research on Cancer (IARC) conducted a review of the published literature and classified RF-EMF as a “possible” (Group 2B) carcinogen for humans^3,4^. The review of health risk studies by The International Commission on Non-Ionising Radiation Protection (ICNIRP) considers the effects of RF-EMF on body systems, processes or specific diseases. Experimental tests on cells (animal and human) and observational studies on humans, that evaluate the interconnection between RF-EMF and a range of potencial health-related effects were described. It is concluded that the only justifiable adverse health effects caused by RF-EMF exposure are nerve stimulation, changes in cell membrane permeability, and effects associated with increased temperature^5^. The degree of temperature increase depends on RF-EMF parameters, specific absorption coefficient, time parameters, and localization of exposure. The physiological response of the body was studied in great detail in this document.

However, body weight, which is another informative physiological response of an experimental animals’ body to any exposure, is studied much more poorly. It is an objective measure of health and development in laboratory animals^6^, an indicator of stress and distress^7^, and it is used as an objective sign of pain and discomfort^8,9^. Organ mass is also one of the most sensitive indicators of toxicity, and its changes often precede morphological changes^10,11^. Therefore, in this article, a weekly study of the body weight of male Wistar rats of different ages and a study of organ weights after euthanasia under chronic RF-EMF exposure were carried out for the first time.

The study of cognitive reactions in rats exposed to RF-EMF was carried out using a standard method—the Morris water maze test. A large systematic review^12^ provides contradictory results of studies of the RF-EMF effect on the cognitive behavior of laboratory animals. The authors believe that the contradictions stem from the lack of detailed dosimetry provided by heterogeneous, anatomically realistic animal models. Narayanan et al. showed a violation of the spatial navigation ability in animals exposed to RF-EMF, compared to the control group. This can be caused by the effect of RF-EMF on glial cells and the modulation of neurotransmitter levels in different brain areas^13,14^. Structural changes, which may occur in various areas of the brain (blood–brain barrier, hippocampal formations, cerebral cortex, cerebellum, and amygdala), were described^15^.

This study aims to investigate (for the first time) the effect of chronic exposure to radiofrequency radiation from the laboratory base station 5G NR on basic physiological parameters and cognitive abilities of male Wistar rats of different ages—juvenile, adult, and presenile. These age groups were studied because they have different levels of metabolism and, therefore, studied parameters may differ.

## Results

### The body weight in the dynamics of the experiment

In the dynamics of the experiment, the body weight of Wistar rats did not show statistically significant differences in the experimental rats exposed to RF-EMF 5G NR compared to control rats of the corresponding ages. The results obtained when measuring the body weight of male Wistar rats of the studied groups are presented in Table 1.

**Table 1:** Body weight (g) of Wistar rats of the studied ages during the experiment.

Group of animals	Prior to the experiment	1 week	2 week	Week 3	Week 4	5 weeks (After testing in the MWM)
		Exposure to RF-EMF 5G				
Juvenile rats						
Control (n = 10)	227.4 ± 15.8	273.8 ± 21.2	298.7 ± 23.9	316.5 ± 27.6	328.2 ± 27.9	338.3 ± 28.2
exposure to RF-EMF 5G (n = 10)	232.7 ± 20.6 *p* = 0.108	280.1 ± 22.2 *p* = 0.068	299.7 ± 18.7 *p* = 0.593	313.3 ± 24.0 *p* = 0.108	327.0 ± 25.6 *p* = 0.361	344.3 ± 27.0 *p* = 0.068
Adult rats						
Control (n = 10)	260.2 ± 18.9	298.2 ± 22.2	317.4 ± 26.5	335.7 ± 26.7	348.3 ± 25.9	346.4 ± 27.8
exposure to RF-EMF 5G (n = 10)	273.3 ± 20.7 *p* = 0.068	314.5 ± 30.0 *p* = 0.068	331.1 ± 30.6 *p* = 0.068	347.3 ± 33.9 *p* = 0.068	355.4 ± 31.5 *p* = 0.138	368.3 ± 33.5 *p* = 0.068
Presenile rats						
Control (n = 10)	495.1 ± 55.1	495.0 ± 62.1	497.3 ± 61.4	498.1 ± 64.2	513.2 ± 54.1	519.2 ± 84.2
exposure to RF-EMF 5G (n = 10)	488.0 ± 43.9 *p* = 0.068	488.5 ± 48.0 *p* = 0.273	490.5 ± 45.8 *p* = 0.273	493.9 ± 49.3 *p* = 0.273	509.9 ± 54.5 *p* = 0.068	493.4 ± 51.4 *p* = 0.225

The data are presented as an average value ± standard error. Statistical analysis was carried out using the Statistica 6.0 software package (StatSoft). The nonparametric Mann–Whitney test was used to verify the reliability of group differences between the experimental and control groups of rats. The *p*-value is given in each age group compared to the control group. The differences are statistically significant at *p* < 0.05.

### The weight coefficients of internal organs

The weight coefficients of internal organs after exposure to RF-EMF in rats of the studied ages were determined after the animals were sacrificed. No statistically significant differences between exposed and control rats were detected (Table 2).

**Table 2:** The weight coefficients of internal organs (% of body weight) of male rats after a five-week-long exposure to 5G RF-EMF.

Group of animals	Brain	Heart	Liver	Left testicle	Right testicle
Juvenile rats					
Control (n = 10)	0.53 ± 0.03	0.26 ± 0.01	4.81 ± 0.42	0.54 ± 0.06	0.48 ± 0.07
exposure to RF-EMF 5G (n = 10)	0.54 ± 0.05 *p* = 0.068	0.29 ± 0.08 *p* = 1.000	4.22 ± 0.74 *p* = 0.068	0.59 ± 0.08 *p* = 0.068	0.58 ± 0.09 *p* = 0.068
Adult rats					
Control (n = 10)	0.51 ± 0.04	0.22 ± 0.05	4.54 ± 0.18	0.50 ± 0.06	0.50 ± 0.07
exposure to RF-EMF 5G (n = 10)	0.52 ± 0.12 *p* = 0.575	0.23 ± 0.02 *p* = 0.273	4.73 ± 0.34 *p* = 0.273	0.55 ± 0.05 *p* = 0.068	0.56 ± 0.04 *p* = 0.108
Presenile rats					
Control (n = 10)	0.35 ± 0.06	0.24 ± 0.02	3.33 ± 0.64	0.43 ± 0.05	0.43 ± 0.05
exposure to RF-EMF 5G (n = 10)	0.38 ± 0.02 *p* = 0.144	0.25 ± 0.02 *p* = 0.179	3.42 ± 0.57 *p* = 0.068	0.44 ± 0.02 *p* = 0.273	0.45 ± 0.01 *p* = 0.273

The data is presented as an average value ± standard error. Statistical analysis was carried out using the Statistica 6.0 software package (StatSoft). The nonparametric Mann–Whitney test was used to verify the reliability of group differences between the experimental and control groups of rats. The *p*-value is given in each age group compared to the control group. The differences are statistically significant at *p* < 0.05.

### The rectal and skin surface temperature

In the course of the experiment, the rectal and skin surface temperature of the male experimental 5G rats did not show significant differences from the indicators of the control rats of the corresponding ages. The data obtained are presented in Tables 3 and 4.

**Table 3:** The rectal temperature (°C) of rats during the experiment.

Group of animals	Prior to the experiment	1 week	2 week	Week 3	Week 4
Juvenile rats					
Control (n = 10)	37.6 ± 0.6	37.5 ± 0.3	37.5 ± 0.4	37.9 ± 0.3	37.7 ± 0.2
exposure to RF-EMF 5G (n = 10)	37.4 ± 0.6 *p* = 0.108	37.6 ± 0.5 *p* = 0.068	37.3 ± 0.3 *p* = 0.068	37.6 ± 0.3 *p* = 0.068	37.4 ± 0.4 *p* = 0.068
Adult rats					
Control (n = 10)	37.5 ± 0.4	37.7 ± 0.4	37.4 ± 0.5	37.8 ± 0.3	37.5 ± 0.3
exposure to RF-EMF 5G (n = 10)	37.7 ± 0.3 *p* = 0.068	37.8 ± 0.4 *p* = 0.068	37.3 ± 0.5 *p* = 0.068	37.4 ± 0.6 *p* = 0.068	37.8 ± 0.3 *p* = 0.068
Presenile rats					
Control (n = 10)	36.8 ± 0.7	36.4 ± 0.4	36.3 ± 0.5	36.8 ± 0.6	37.3 ± 0.4
exposure to RF-EMF 5G (n = 10)	36.4 ± 0.8 *p* = 0.108	37.0 ± 0.6 *p* = 0.179	36.0 ± 0.7 *p* = 0.068	37.1 ± 0.6 *p* = 0.108	37.5 ± 0.5 *p* = 0.361

The data are presented as an average value ± standard error. Statistical analysis was carried out using the Statistica 6.0 software package (StatSoft). The nonparametric Mann–Whitney test was used to verify the reliability of group differences between the experimental and control groups of rats. The *p*-value is given in each age group compared to the control group. The differences are statistically significant at *p* < 0.05.

**Table 4:** The temperature (°C) of the skin surface of rats during the experiment.

Group of animals	Prior to the experiment	1 week	2 week	Week 3	Week 4
Juvenile rats					
Control (n = 10)	35.8 ± 0.4	35.3 ± 0.6	36.3 ± 0.2	36.1 ± 0.1	36.2 ± 0.2
exposure to RF-EMF 5G (n = 10)	35.8 ± 0.6 *p* = 1.000	35.8 ± 0.5 *p* = 0.068	36.3 ± 0.3 *p* = 0.422	36.1 ± 0.1 *p* = 0.179	36.3 ± 0.1 *p* = 0.108
Adult rats					
Control (n = 10)	36.1 ± 0.2	36.1 ± 0.2	36.0 ± 0.3	36.0 ± 0.3	36.1 ± 0.1
exposure to RF-EMF 5G (n = 10)	36.0 ± 0.2 *p* = 0.067	36.1 ± 0.5 *p* = 0.592	36.1 ± 0.2 *p* = 0.108	36.0 ± 0.2 *p* = 0.422	36.2 ± 0.2 *p* = 0.422
Presenile rats					
Control (n = 10)	36.3 ± 0.2	36.2 ± 0.4	36.2 ± 0.1	36.0 ± 0.3	36.1 ± 0.1
exposure to RF-EMF 5G (n = 10)	35.7 ± 0.9 *p* = 0.144	36.2 ± 0.1 *p* = 0.422	36.1 ± 0.2 *p* = 0.108	36.1 ± 0.2 *p* = 0.108	36.2 ± 0.2 *p* = 0.422

The data are presented as an average value ± standard error. Statistical analysis was carried out using the Statistica 6.0 software package (StatSoft). The nonparametric Mann–Whitney test was used to verify the reliability of group differences between the experimental and control groups of rats. The *p*-value is given in each age group compared to the control group. The differences are statistically significant at *p* < 0.05.

### A study of the cognitive functions of Wistar rats of the studied age groups in the Morris water maze (MWM)

**Figure 1:**
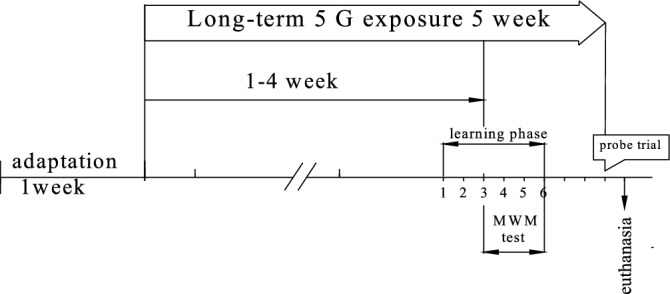
Experimental design and schedule for the Morris water maze test. The "learning phase" is training time, which includes 1 day of training/getting used to the water maze environment (swimming in water) and 4 days of training aimed at finding the hidden platform. The "probe trial" is a test of acquired memory.

Experimental design and schedule for the Morris water maze test shown in Fig. 1.

A study of the cognitive functions of Wistar rats of the studied age groups in the Morris water maze (MWM) also showed no statistically significant differences between the experimental and control groups of rats. The results are presented in Table 5 and Figure.

**Table 5:** Testing of Wistar rats of the studied age groups in the MWM (N = 10).

Average time of successful swims (in seconds)						
Age	Series	1 day	Day 2	Day 3	Day 4	Day 7
Testing days						
Juvenile	Control (n = 10)	28.9 ± 15.4	21.1 ± 8.0	15.8 ± 3.3	19.5 ± 9.5	21.5 ± 7.0
	Exposure to RF-EMF 5G (n = 10)	27.3 ± 8.3 *p* = 0.465	21.7 ± 7.2 *p* = 0.715	19.2 ± 4.4 *p* = 0.068	18.3 ± 5.9 *p* = 0.715	21.3 ± 8.2 *p* = 0.715
Adult	Control (n = 10)	22.2 ± 8.2	23.9 ± 10.4	17.7 ± 7.7	15.9 ± 6.1	17.0 ± 9.5
	Exposure to RF-EMF 5G (n = 10)	25.8 ± 9.6 *p* = 0.068	22.7 ± 8.6 *p* = 0.465	17.5 ± 6.1 *p* = 0.273	14.8 ± 5.5 *p* = 0.144	13.2 ± 4.9 *p* = 0.144
Presenile	Control (n = 10)	37.7 ± 9.5	22.4 ± 6.0	21.8 ± 6.3	16.4 ± 7.5	16.9 ± 7.1
	Exposure to RF-EMF 5G (n = 10)	30.4 ± 9.1 *p* = 0.068	23.1 ± 5.2 *p* = 0.144	21.1 ± 11.1 *p* = 0.592	16.9 ± 5.3 *p* = 0.465	15.6 ± 3.7 *p* = 1.000
Percentage of successful swims						
Juvenile	Control (n = 10)	55.5	63.9	81.2	83.3	90.6
	Exposure to RF-EMF 5G (n = 10)	63.6	86.4	93.2	97.7	90.0
Adult	Control (n = 10)	65.0	92.5	92.5	100	100
	Exposure to RF-EMF 5G (n = 10)	65.9	90.0	93.2	97.7	95.4
Presenile	Control (n = 10)	59.1	81.8	95.4	95.4	95.4
	Exposure to RF-EMF 5G (n = 10)	66.7	85.4	91.7	95.8	100

The data are presented as an average value ± standard error. Statistical analysis was carried out using the Statistica 6.0 software package (StatSoft). The nonparametric Mann–Whitney test was used to verify the reliability of group differences between the experimental and control groups of rats. The *p*-value is given in each age group compared to the control group. The differences are statistically significant at *p* < 0.05.

The overall dynamics of ethology in MWM animals of all studied age groups corresponded to the norm: acceleration of time of successful swims and an increase in the percentage of successful swims from the 1st to the 4th day of testing. Juvenile rats of the control group and the experimental group (exposed to RF-EMF 5G) showed better performance (average swim time) on the first day of testing compared to similar groups of presenile animals, but on the 7th day of testing their performance was slightly lower than that of the adult and presenile rats.

**Figure 2:**
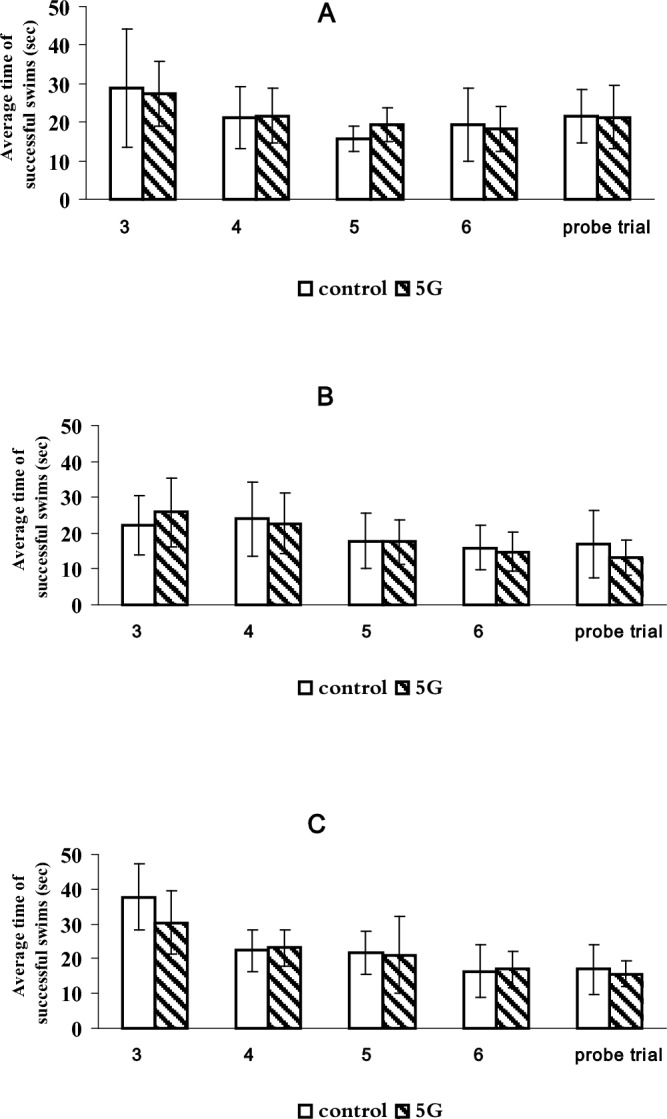
Spatial learning and memory during the training days in the Morris water maze in different groups: (**A**) juvenile rats; (**B**) adult male rats; (**C**) presenile male rats.

Since the MWM is effective when detecting deviations in memory and learning functions, we consider it possible to assume that juvenile Wistar rats learn faster than presenile rats, but memory is preserved better in adult and presenile rats than in juveniles. There were no differences in the performance of ethology in MWM between the control rats and the rats exposed to RF-EMF 5G in each age group (Fig. 2).

Previously, it was found that “about 20% of the variability is explained by the different propensity of rats to swim passively in the water until the experimenter ‘saves them’”^16^. Therefore, in Wistar rats, the tags on their ears (the first digit is the cell number, the second is the individual rat number) helped to determine which animals were inclined to passive swimming when testing in MWM (Table 6).

**Table 6:** The numbers of Wistar rats of the studied age groups that performed passive swimming (more than 60 s) while being tested in the Morris pool.

Testing days						
Age	Series	Day 1	Day 2	Day 3	Day 4	Day 7
Juvenile	Exposure to RF-EMF 5G (n = 10)	1–0 1–8 1–6	1–8			
	Control (n = 10)	2–4 2–6 2–9 2–3	2–4 2–6 2–9 2–3	2–4 2–3	2–4	2–4
Adult	Exposure to RF-EMF 5G (n = 10)	3–10 3–8 3–2 3–5	3–8			
	Control (n = 10)	4–0 4–4 4–3 4–7 4–1	4–0			
Presenile	Exposure to RF-EMF 5G (n = 10)	4–5 4–0 2–0	2–0	4–5		
	Control (n = 10)	3–4 3–0 1–4 1–1 1–0	3–0 1–0			3–0

An interesting fact is that most episodes of passive swimming took place on the 1st day of testing, after the first or second attempt. Some rats, mainly juvenile control rats, maintained this tendency to swim passively in the following days of testing. However, juvenile experimental rats "rejected" passive swimming faster in comparison with the corresponding control rats. This may also indicate that RF-EMF contributes to better training of juvenile Wistar rats. It is worth noting that during long periods of passive swimming, the animals' fur gets wetter and it requires a massive effort from the rat to keep on the water surface. On the 3rd day of testing, all adult rats rejected passive swimming, and one presenile control rat was adrift even on the 7th day. Apparently, these differences are related to the animals' age and their individual differences in assessing the surrounding circumstances and choosing a behavior strategy.

## Discussion

This article presents the research results of weight and body temperature parameters of Wistar rats and their cognitive abilities during long-term exposure to radiofrequency electromagnetic field from the new generation base station 5G NR. Despite the importance of weight parameters as indicators of body condition, such data are poorly represented in the research literature. We studied basic weight parameters in rats of three ages (juvenile, adult, and presenile) that differ in metabolic levels. The absence of statistically significant differences in rats exposed to RF-EMF 5G compared to control (not exposed) animals was found in all studied weight and temperature parameters, and in test results in the Morris water maze.

It was found that there were no statistically significant changes in body weight in the rats exposed to RF-EMF 5G compared to control animals of the same age (Table 1). Table 1 shows that during the five-week experiment in juvenile rats, the body weight gain decreased and ranged from ~ 46–48 g in the 1st week to ~ 10–17 g in the last. In adult rats, the weekly increase in body weight also decreases (but it is less pronounced), and in presenile animals, this indicator almost does not change during the five-week experiment. It is obvious that the decrease in body weight gain is naturally determined by the increase in the age of animals during the experiment^6,17^.

However, some differences were still observed. All rats of all ages were provided with more than sufficient feed and water in standard plastic bottles of 800 ml was changed daily. The sawdust litter in the rat cages was changed once a week. Animals of all age groups were kept in the same conditions. But juvenile rats exposed to RF-EMF 5G showed some peculiarities in feed and water intake during the experiment. Thus, starting from Week 3, their feed and water intake was higher than in control juvenile rats and rats of other ages in both experimental (exposed to RF-EMF 5G) and control groups. This increase in exposed to RF-EMF 5G juvenile rats began with a higher feed and water intake by ~ 1.5 times (at Week 3) and up to ~ 2.5 times by the end of Week 5. They also ate significantly more feed than control juvenile rats. Consequently, the sawdust litter in their cages had to be changed not once, but twice a week. Our experiment was designed not to take into account water and food consumption. Nevertheless, in our experiment, the difference in water and feed consumption between juvenile rats, and juvenile control rats was so great that it was impossible not to notice it. In addition, the body weight of juvenile rats exposed to RF-EMF 5G did not differ from that of control juvenile rats (Table 1).

In the available literature, it was possible to find relatively few studies where mice body weight was determined when they were exposed to RF-EMF. For example, Sommer et al.^18^ show that exposure to GSM-modulated EMFs caused an increase in body weight gain in female adult AKR/J mice compared to control animals. The round-the-clock effect of GSM-modulated fields with a frequency of 900 MHz was studied for 41 weeks, with a whole-body SAR (specific absorption rate) of 0.4 W/kg. And vice versa, de Jenlis et al.^19^ note hypophagia in juvenile rats (aged 3 weeks) that received only RF-EMF (continuous RF-EMF, 900 MHz, 1.8 V/m, SAR = 30 mW/kg). The authors found that exposure to RF-EMF caused a decrease in the total amount of ingested food, and the eating pattern changed (the number of meals and their duration were taken into account). In addition, RF-EMF resulted in greater water consumption than in the control group. There were no discrepancies regarding water consumption in our experiments, but it is important that in the experiments of de Jenlis et al.^19^ it is noted that juvenile rats exposed to RF-EMF gained body weight much faster than control animals without such exposure. This discrepancy might be explained by the fact that they used significantly younger animals in their five-week-long experiment. At the beginning of the studies, the male Wistar rats were three weeks old with a significant variation in initial body weight from 55 to 85 g. At the end of the experiment, the body weight gain (relative to the weight on the day of arrival) in the control rats was 210.5 ± 25.5 g, and in the RF-EMF-exposed rats, it was 231.95 ± 27.6 g, *p* < 0.001^19^. During the same five-week-long experiment, we used male Wistar rats aged 5–6 weeks with an initial body weight of 229 ± 30 g. At the end of the experiments, the juvenile control rats weighed 338.3 ± 28.2 g, and the juvenile experimental 5G rats weighed 344.3 ± 27.0 g. The rapid growth rate of body weight in the experiments of de Jenlis et al.^19^ might have been caused by the fact that their animals were initially younger. However, it is difficult to explain the paradox of the rapid growth rate of body weight under hypophagia. The authors believe that this could have been caused by a decrease in energy consumption due to sleep disorders, when the periods of REM sleep decrease, and a decrease in food intake.

It should be noted that in the experiments of de Jenlis et al.^19^, a higher level of SAR 0.03 W/kg and a lower frequency of 0.9 GHz was used, which differs from the parameters used in our experiments where SAR_1_ was 0.0076 W/kg, SAR_2_ was 0.0059 W/kg_,_ and the frequency was 2.4 GHz. These parameters might also have influenced the discrepancy in the results, since they can have an impact on the studied indicators.

However, the opposite paradox was also established in our experiments. It showed the absence of an increase in body weight under hyperphagia in juvenile animals exposed to RF-EMF 5G. It is assumed that this could be caused by the following mechanisms:

- functional disorders can be due to the influence of RF-EMF on the metabolism and transport of neurotransmitters ^20^. Consequently, RF-EMF can disrupt the neural connections that exist at many brain levels to control eating behavior in accordance with energy homeostasis.
- It is assumed that the sensitivity of neural connections to 5G RF-EMF in the brains of juvenile male rats is higher than in adult and presenile rats, which could affect their eating behavior.
- In our experiments in juvenile rats, exposed to RF-EMF 5G, some manifestations of inflammatory processes in the intestine were detected during autopsy, i.e. bloating of the intestine and unformed stools.
- It is assumed that this could be caused by the vulnerability of the digestive tract in animals that have not reached puberty as a result of hypoplasia of the mucus barrier of the digestive tract^21^.

Thus, the obtained results show that RF-EMF of 2.4 GHz frequency with whole-body SAR values below the basic ICNIRP restrictions does not cause changes in body weight, the most important indicator of physiological state, in male rats of the studied ages, exposed to RF-EMF 5G, compared to control animals. The results obtained on juvenile animals exposed to RF-EMF 5G that also showed no differences in body weight compared to control juvenile rats with marked hyperphagia and increased water intake are of some concern.

Organ mass is one of the most sensitive indicators of toxicity, and the changes in organ mass often precede morphological changes^10,11^. In our experiments, the weight coefficients of the studied organs and the animals’ body weight generally aligned with indicators for the corresponding ages of the studied animals^6,11,17^. There were no statistically significant changes in the weight coefficients of the studied organs in rats after exposure to RF-EMF 5G compared to control animals of the corresponding ages. As follows from Table 2, the brain weights of presenile male Wistar rats were slightly lower than in juvenile and adult animals. Slightly lower values of the weight coefficients of the testicles and liver were also found in presenile rats, compared to juvenile and adult male rats. Apparently, these differences are associated with the higher body weight of presenile animals. In our experiments, no changes in this indicator were obtained in rats of all the studied ages when exposed to 5G radio frequency RF-EMF compared to control animals. The available literature is known to rarely or never indicate any information about body weight and individual organs. Our data might be among the first to describe the state of body weight and internal organs of Wistar rats under the chronic exposure to RF-EMF of 2.4 GHz. However, these indicators are of very high significance not only to assess the condition of animals but also to provide more reliable extrapolation of the results obtained on animals to the human condition.

The core and the body surface temperatures in rats are dependent on the ambient temperature. In our study, well-established methods of measuring rectal and skin surface body temperature were used. The rats were kept in ordinary laboratory cages and were unrestrained. With weekly repetition, the rats get used to temperature measurement procedures and with confident operators using the method of measuring rectal or skin surface temperature, the stress of restriction lasts for several seconds. Measurement of the skin surface temperature (on the upper thigh of the right hind limb) revealed no changes in the dynamics of the experiment from the beginning to the 4th week, nor when exposed to RF-EMF 5G compared to the control animals, nor when different age groups were compared. The skin surface temperature is about 0.5–1.5 °C lower than the core temperature of the body. This corresponds to the previously obtained data from Kawakami et al.^22^, who found that the mouse body surface temperature is about 2.0 °C lower than when measured by a rectal sensor.

There were no significant differences in rectal and skin surface temperature in the experimental 5G rats, compared to the corresponding indicators in the control rats in all the studied age groups.

In our experiments, the temperature in the rooms where the animals were kept was constant at 21 ± 2 °C. It was shown previously that thermoregulatory sensitivity RF-EMF in rodents depends on body weight, ambient temperature, and ability to move^23^. They were found to be important factors influencing the SAR threshold to increase large intestine temperature. Thus, for Sprague–Dawley rats at 20 °C, the SAR threshold values for increasing the large intestine temperature are 1.58 W/kg. At 30 °C, the large intestine temperature increases at SAR 0.4 W/kg^24^. In our case, at an ambient temperature of 22 ± 1 °C and a relatively low SAR, in average 0.0076 and 0.0059 W/kg for two groups of non-anesthetized rats respectively, an increase in the core temperature of the body (large intestine temperature) should not be expected when exposed to the chosen RF-EMF parameters.

It is necessary to study the skin surface temperature when exposed to RF-EMF, since these waves are absorbed within a few millimeters of the skin (depending on wavelength)^5^, and, therefore, the skin is the main target. In a study by Al-Chalabi et al.^25^, it was concluded that a thirty-day-long exposure to LTE (Long-Term Evolution or 4G LTE) 2600 MHz at SAR of 0.982 W/kg for 2 h a day causes an increase in skin temperature in rats. An increase in skin temperature on the back by 1.8 ± 0.3 °C can cause oxidative stress, inflammatory reactions, and significant metabolic changes in the skin of rats^26^. The parameters of RF-EMF used in our study did not lead to an increase in skin surface temperature on the right hind limb in the experimental and control rats of all the studied ages. These parameters were chosen according to the ICNIRP guidelines. The lack of statistically significant differences in rectal and skin surface temperature measurements between experimental (exposed to RF-EMF 5G) and control animals may serve as additional knowledge for the established exposure limits.

The study of the cognitive functions of Wistar rats of the studied age groups in the MWM showed no statistically significant differences between the experimental group exposed to RF-EMF 5G and the control group of rats. This corresponds to the data given in a large literature review on the biological effect of high-frequency RF-EMF on Wistar rats. The authors note that “although microwave radiation of 2.45 GHz caused harmful changes in the brain leading to a decrease in learning and memory, severity of anxiety behavior in rats, and crash of antioxidant enzyme systems of the brain, no significant difference was observed in the MWM”^27^. Other authors have also shown the opposite effect: reduced spatial navigation in rats exposed to a mobile phone^28^. The changes in spatial memory may be associated with the effect on markers of oxidative stress^29^ and neuroplasticity^30^. In our experiment, tests in the MWM revealed some differences in the effects of exposure to RF-EMF 5G in animals of different ages. The results obtained on the percentage of successful swims suggest that juvenile Wistar rats learn faster than presenile rats, but memory in adult and presenile rats is preserved better than in juvenile rats.

Thus, the study showed an absence of statistically significant changes in the body weight of the experimental 5G Wistar rats of juvenile, adult, and presenile ages. Since this indicator is an objective measure of health and/or development of laboratory animals, an indicator of animal distress, and is used as an objective sign of pain and discomfort, it can be concluded that the experimental Wistar rats exposed to RF-EMF 5G did not show changes in their condition compared to control rats not exposed to RF-EMF 5G. The core temperature of the body and skin surface temperature remained the same in the experimental 5G rats compared to the control animals of the corresponding age. The absence of thermal effects was due to the implementation of international and Russian regulations for the use of 5G RF-EMF, which do not provide for thermal effects.

In the MWM cognitive test, it was found that juvenile Wistar rats learn faster than presenile rats, but memory in adult and presenile rats is preserved better than in juvenile rats. Additionally, juvenile experimental 5G rats, compared with the corresponding control, “rejected” passive swimming faster, i.e., they chose a different “test execution strategy”. It is assumed that the results obtained can be used to predict the state of a person under chronic exposure to a new generation 5G wireless transmission antenna.

Future studies on the effects of exposure to RF-EMF 5G should be conducted on female Wistar rats as well in order to predict the impact of RF radiation from base stations 5G NR on all groups of the population.

## Materials and methods

### Ethical approval

All procedures with animals were carried out in accordance with the EU Council Directive 86/609/EEC of 24 November 1986 and were approved by the Animal Research Ethics Committee of Tomsk State University. Our study was conducted following the recommendations of ARRIVE^31^. Extract from Record No. 6 of the meeting of the TSU Committee on Bioethics as of 04.04.2022, registration No. 10. Approval for experiments involving animals is based on a harm–benefit analysis, weighing the harms to the animals involved against the benefits of the research to society^31^.

### Experimental animals

Sixty male Wistar rats were raised and provided by the Goldberg Research Institute of Pharmacology and Regenerative Medicine of the Tomsk National Research Medical Center. According to an external clinical examination, the results of laboratory diagnostic studies, and luminescent diagnostics, the rats were clinically healthy: the animals were of ‘normal’ fatness, they moved actively, ate food, drank water, their fur was clean, and there were no mucus secretions. The body weight range in each age group of rats was less than 10%. Vaccination/immunization was not carried out. The area was free from infectious animal diseases. We received 20 juvenile male rats (aged 5–6 weeks), 20 adult male rats (aged 10–11 weeks), and 20 presenile male rats (aged 17–18 weeks). The presenile rats were received in August 2022, and the juvenile and adult rats in September 2022. Thus, their exposure under the RF antenna was carried out sequentially: the presenile rats were the first, then the juvenile and adult rats.

Immediately after receiving the rats, they were randomly distributed into polycarbonate cages (dimensions in mm 480 × 210 × 375) with sawdust litter, free access to water, and standard combined feed for laboratory animals (produced by JSC BioPro, Novosibirsk, Russia). The presenile rats were distributed 5 animals per cage, and juvenile and adult rats were distributed 10 animals per cage. To determine the possible individual dynamics of the studied parameters, after placing all the rats in each cage, they were labeled by numbers from 1 to 10 (for juvenile and adult rats) or from 1 to 5 (for presenile rats). Quarantine lasted 1 week in a room with natural light, ventilation, a temperature of 22 ± 1 °C, and humidity of 45–50%, with unrestricted feed and drinking water. The animals were examined by qualified staff 3–4 times a day. We observed the cleanliness of rats' hair and tail, the absence of any discharges from their eyes and nose, the hygiene of their litter, and the favorable condition of the animals.

### The protocol

On the day after quarantine ended, the rats were divided into the experimental 5G and control groups. The cells with control groups remained in the same room, and the cells with experimental groups were placed in a separate room with similar conditions, where a 5G radio frequency antenna was installed. Table 7 shows the distribution of experimental and control groups of rats in the cage.

**Table 7:** The distribution of experimental and control groups of rats in the cage.

Presenile rats	
Cell 1 (n = 5)—5G	cell 2 (n = 5) – control
Cell 3 (n = 5)—5G	cell 4 (n = 5) – control
Juvenile rats	
Cell 1 (n = 10)—5G	cell 2 (n = 10)—control;
Adult rats	
Cell 3 (n = 10)—5G	cell 4 (n = 10) – control

### Data collection

After a week of quarantine prior to conducting the experiment, the body weight of the rats was determined on a WS-23 scales (Precision Mechanics Plant, 119,821, Poland), the rectal temperature was measured using an A&D rectal electronic thermometer DT-501 (A&D Company Ltd D210565251, Japan), and skin surface temperature on the right hind limb by using a CS Medica KIDS Cs-88 infrared thermometer (Vega Technologies Inc., 4607043670915, Taiwan). Before measuring the skin surface temperature, the hair on the skin area of the right hind limb was shaved off using a Gemmy razor for laboratory animals (China). Weighing and measurements of rectal and skin surface temperature were carried out in the daytime from 10 am to 12 pm. All procedures were performed once a week without anesthesia.

The experimental 5G rats were constantly exposed to RF-EMF 5G for 4 weeks around the clock. Before conducting the experiment and then during every week, the rats were weighed and their rectal and skin surface temperature was measured. Then they were tested for one more week in the MWM (1st, 2nd, 3rd, 4th, and 7th days of testing). The pool of the water maze was located outside the room with an RF-EMF setup. Testing of rats in each cage took from 35 to 60 min; after testing, the experimental groups of rats were returned to the room with a source of RF-EMF. Thus, during the 5th week, there was no round-the-clock exposure to RF-EMF, but during the days of testing in the water maze, there was an exposure with a 60-min break to conduct it.

Animals of control (not exposed) groups were placed in the same maintenance conditions (temperature, humidity, cleaning, and feeding regime) in another room, where the density of the EMF energy flow corresponded to the background RF-EMF level, at a 30 m distance from the room with the installed antenna.

Before conducting the experiment and then during every week, body weight, rectal, and skin surface temperature were also determined in the control rats (for 4 weeks), and then they were tested during a week in the MWM.

At the end of the experiment, all the rats were weighed the next day after testing in the MWM was completed (5 weeks after the start of the experiment). Their organs were isolated: brain, heart, liver, and right and left testicles. The organs were weighed on A&D GR-120 electronic scales (A&D Company Ltd 14227755, Japan). The weight coefficients of the internal organs were determined as a percentage of the body weight on the same day.

### The 5G radiation setup and dosimetry

**Figure 3:**
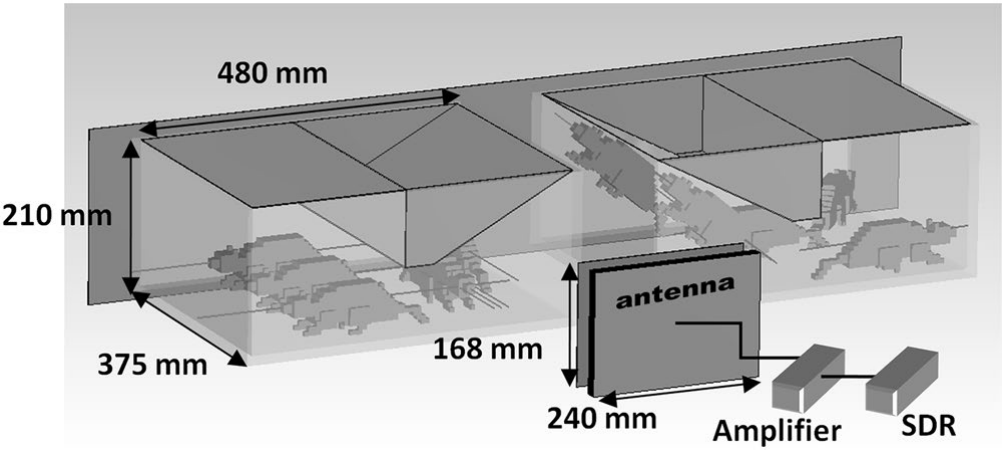
The Exposure setup.

**Figure 4:**
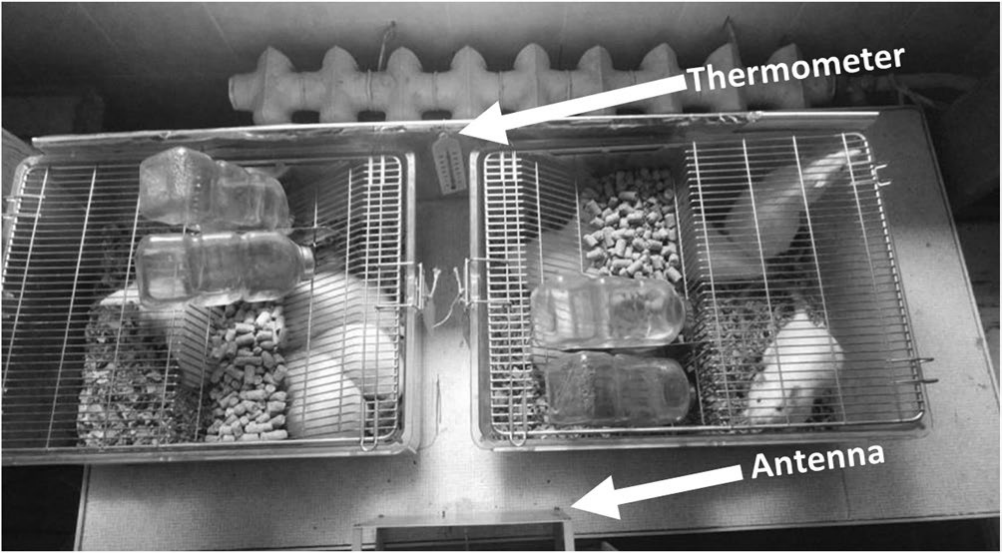
A photograph of the experiment.

The laboratory setup for radiating the 5G RF-EMF comprised a power source, a software-defined radio, an amplifier, and a single combined antenna (Figs. 3 and 4).

An ADALM-Pluto (Analog Devices, Inc. One Technology Way, Norwood, MA 02062-9106, USA. Manufactured in PRC.) Software Defined Radio (SDR) is configured to cyclically generate one frame of the 5G NR downlink in the n40 (2.4 GHz) operating range, which is defined according to the table of the 3GPP TS38.104 standard European Telecommunications Standards Institute ^32^.

The bandwidth of the generated signal is 15 MHz. The signal simulates the transmission of user data and control information from the base station. A cascade amplifier on the blm9D2324-25b (Ampleon, 9349 600 73515, Netherlands B.V.) board with a gain of 28 dB in the operating frequency range is used to amplify the signal. A combined antenna with dimensions of 240 × 168 mm and a gain of ~ 7 dB is connected to the output of the cascade amplifier. The resulting power at the antenna input was 0.0891 W. The use of a combined antenna, which is an electric and magnetic dipole with a combined phase center, implies that the far field is formed at a distance of the wavelength of the radiation used (~ 12 cm).

There are a number of experimental methods for determining SAR, including waveguide, calorimetric, thermometric methods, and the methods based on the measurement of EMF components in biomaterials, or the use of personal exposimeters^33^. These methods are not always applicable because they require specialized equipment and have certain limitations when dosimetry is used to measure radiation absorbed by living organisms. Therefore, in a real-life setting, it is not always feasible to accurately determine SAR.

For the current animal irradiation configuration, it can be observed that the incident power density of the electromagnetic radiation decreases with distance from the antenna. And the SAR estimation with this kind of setup should be calculated considering the distance from the antenna. Therefore, the co-authors conducted a study on SAR estimation for this experiment^34^. For this configuration, the whole-body SAR estimate is calculated as

1 $$ SAR_{wb.avg} \approx \frac{5.954}{{f_{GHz} }}\frac{{\left\langle {S_{inc} (r)} \right\rangle \left\langle L \right\rangle }}{\left\langle m \right\rangle } $$

where < *m* > is the averaged mass of the animals in kilograms, < *L* > – the length of the animals averaged in meters. $S_{inc} (r) = P_{A} G/4\pi r^{2}$—incident power density, $P_{A}$ is the power supplied to the antenna [W], *G* is the antenna gain [dB], *r* is the distance from the antenna [m]. $< S_{inc} (r) >$ is the averaged incident power density over *r*.

To validate the SAR estimations from Eq. 1, a numerical simulation of an experiment for SAR calculation in primitive rat models was carried out in the CST Microwave Studio software environment. The values of the dielectric parameters in the numerical model were used from the library of the software environment. The IEEE/IEC 62704-1 algorithm was used to calculate SAR in the numerical model. The presence of metallic surfaces near the animals may affect the resultant field in the animal area and consequently contribute to the absorption. For the present experiment geometry, taking reflections and multiple scattering into account, calculating the SAR seems to be a challenging task. In estimating the SAR for this setup, reflections and multiple scattering were not taken into account assuming that they make a small contribution to the absorption. A detailed description of antenna design, its characteristics, dosimetry study, and validation results of the SAR dosimetry in this experiment is provided in^34^.

Prior to the experiment, the background incident EMF power density level was measured in the rooms with the cages at the frequency range used. This background radiation could be emitted from external sources, such as Wi-Fi, cell towers, etc. Measurements were made at different points in the rooms (room with control cages and room with 5G cages), with a microwave power meter M3M-18 (Micran Joint Stock Company, 36974-08, Russia). In both rooms, the incident power density averaged 0.000498 W/m2, which is qualitatively comparable to the results of^35^ in the worldwide tendency. During the experiment, with the antenna switched on, the level of power density (averaged over 30 min) in the room with control animals was also checked. It remained at the same background level for the control room. At a distance of ~ 12 cm from the antenna in the direction of cells, the incident power density was 2.5 W/m2 (averaged over 30 min). All background measurements were provided only for 2.4 GHz frequency.

ICNIRP defines an acceptable whole body SAR value = 0.08 W/kg for exposure times up to 30 min regardless of radiation frequency. In the setup, the SAR value varied from 0.0322 to 0.0017 W/kg, which is approximately several times less than determined by ICNIRP; however, the exposure time was 24 h a day for 4 weeks and one more week (the 5th) with breaks of up to 60 min for testing in the MWM. For each group, the mean SAR value was calculated, from all possible absorptions in the cage region. Also, according to Table 5 in^5^, it is worth noting that for the 2.4 GHz band used, there are other reference levels (averaged over 30 min) that provide an equivalent level of protection. For whole-body irradiation, the incident power density is limited to 10 W/m2, which is ~ 4 times higher than the maximum possible in this experiment.

Thus, in this study, the exposure to RF-EMF corresponds to the ICNIRP guidelines for limiting the impact of the EMF (from 100 kHz to 300 GHz)^5^ and models the impact of the 5G transmitting antenna on the public in accordance with the norms established in the Russian Federation^36^. These standards are established for the use of radio-electronic means of fifth-generation communication networks within the pilot zones in order to conduct research, experimental, and design works at their location.

### Operating modes of the laboratory setup

The first exposure was carried out by installing 2 cells at a distance of 12 cm from the antenna (cells N1 (n = 5) and N3 (n = 5) with presenile rats), the average SAR value was ~ 0.0076 W/kg.

The second exposure was carried out by installing 2 cells at a distance of 20 cm from the antenna (cell N1 (n = 10) with juvenile rats and cell N3 (n = 10) with adult rats), the average SAR value was ~ 0.0059 W/kg.

Two exposure levels (0.605 and 0.31 W/m2) were due to different body weights and body lengths of rats of different ages. Taking into account the fact that the rats were located in the cages and unrestrained, the SAR value in the first exposure (for presenile rats) ranged from 0.0322 (max $S_{inc}$ = 2.5 W/m2) to 0.0017 (min $S_{inc}$ = 0.13 W/m2), and 0.0076 W/kg on average ($< S_{inc} (r) >$  = 0.605 W/m2). The SAR value in the second exposure (for juvenile rats and adult rats) ranges from 0.0153 (max $S_{inc}$ = 0.91 W/m2) to 0.0022 (min $S_{inc}$ = 0.13 W/m2), with an average of 0.0059 W/kg (average $< S_{inc} (r) >$  = 0.31W/m2). The SAR value decreases as the rats move away from the irradiating antenna. Such operating modes were chosen specifically to avoid exceeding ICNIRP norms and also to avoid a temperature rise in the animals.

### The Morris Water Maze (MWM)

After preliminary training of rats, testing was carried out according to the standard methodology^37^ using a pool with a diameter of 1.5 m and a height of 0.6 m, with a hidden platform (10 cm in diameter) and three different geometric objects on the walls of the pool used as landmarks. The location of these landmarks and the starting point were always the same. The water temperature in the pool was 22 ± 1 °C. Each training test lasted 60 s. The test time was recorded using a stopwatch. The time of a successful swim (how many seconds it took for the rat to find a hidden platform) was determined, and the average time of successful swims for each group of rats was calculated on each day. Then the percentage of successful swims was calculated. If an attempt failed and a rat did not find the hidden platform within 60 s, the animal was placed on the platform for 10–15 s. Each rat was subjected to four training tests per day for 4 days (1st, 2nd, 3rd and 7th days). The testing procedure in the MWM was carried out from 10 am to 1 pm. The water in the pool was changed after each day of testing.

### Statistical analysis

The data are presented as an average value ± standard error. Statistical analysis was performed using the software package STATISTICA 6.0 (Stat Soft, Inc., USA). The threshold of statistical significance was set at *p* ≤ 0.05.

## Data Availability

All raw data and photos of experimental procedures were uploaded to Figshare (10.6084/m9.figshare.23634618).
